# RNAi, DRD1, and Histone Methylation Actively Target Developmentally Important Non-CG DNA Methylation in *Arabidopsis*

**DOI:** 10.1371/journal.pgen.0020083

**Published:** 2006-06-02

**Authors:** Simon W.-L Chan, Ian R Henderson, Xiaoyu Zhang, Govind Shah, Jason S.-C Chien, Steven E Jacobsen

**Affiliations:** 1 Department of Molecular, Cell, and Developmental Biology, University of California Los Angeles, Los Angeles, California, United States of America; 2 Howard Hughes Medical Institute, University of California Los Angeles, Los Angeles, California, United States of America; University of Wisconsin, United States of America

## Abstract

Cytosine DNA methylation protects eukaryotic genomes by silencing transposons and harmful DNAs, but also regulates gene expression during normal development. Loss of CG methylation in the *Arabidopsis thaliana met1* and *ddm1* mutants causes varied and stochastic developmental defects that are often inherited independently of the original *met1* or *ddm1* mutation. Loss of non-CG methylation in plants with combined mutations in the *DRM* and *CMT3* genes also causes a suite of developmental defects. We show here that the pleiotropic developmental defects of *drm1 drm2 cmt3* triple mutant plants are fully recessive, and unlike phenotypes caused by *met1* and *ddm1,* are not inherited independently of the *drm* and *cmt3* mutations. Developmental phenotypes are also reversed when *drm1 drm2 cmt3* plants are transformed with *DRM2* or *CMT3,* implying that non-CG DNA methylation is efficiently re-established by sequence-specific signals. We provide evidence that these signals include RNA silencing though the 24-nucleotide short interfering RNA (siRNA) pathway as well as histone H3K9 methylation, both of which converge on the putative chromatin-remodeling protein DRD1. These signals act in at least three partially intersecting pathways that control the locus-specific patterning of non-CG methylation by the DRM2 and CMT3 methyltransferases. Our results suggest that non-CG DNA methylation that is inherited via a network of persistent targeting signals has been co-opted to regulate developmentally important genes.

## Synopsis

The majority of DNA in large eukaryotic genomes (such as the human genome) consists of transposons, sequences that can reproduce at the expense of their host. Plants and animals mark transposon DNA with a chemical modification called DNA methylation. DNA methylation prevents the functional information in transposons from being copied into RNA and utilized—this process is termed “gene silencing.” Using a flowering plant called *Arabidopsis,* the authors created mutants lacking a particular type of DNA methylation, and found that these plants had defects in leaf shape, plant height, and fertility. This shows that a gene-silencing mechanism used to defend the genome from transposons is also important for normal plant development. When the mutated genes are restored, plant development returns to normal, showing that one type of DNA methylation can be efficiently re-established (other gene-silencing marks can be lost irreversibly). Small RNA molecules are important for targeting DNA methylation to transposons and harmful DNAs. Mutants in genes that are important for making small RNAs have similar developmental defects to those lacking DNA methylation. This implies that normal plant development requires DNA methylation that is targeted by small RNAs.

## Introduction

The *met1* and *ddm1* mutations that affect maintenance of CG DNA methylation cause severe and variable developmental defects, suggesting that DNA methylation can affect many developmental genes [1–4]. *MET1* encodes a maintenance DNA methyltransferase orthologous to mouse Dnmt1, and *DDM1* encodes a SNF2-like chromatin-remodeling ATPase [5,6]. Some of the developmental phenotypes in *met1* and *ddm1* clearly result from loss of CG DNA methylation at particular genes. In the case of the *FWA* gene, loss of CG DNA methylation in *met1* causes overexpression of the FWA transcription factor, resulting in a dominant and heritable late-flowering phenotype [3,4,7]. Unmethylated *fwa* segregates as an independent trait because CG DNA methylation is not regained when *met1* is crossed to wild type. An independently segregating developmental phenotype caused by loss of CG DNA methylation has also been observed at the *BAL* locus, which encodes a pathogen-resistance gene within a repetitive gene cluster [8].

DNA methylation is found at cytosines in three different sequence contexts, CG, CNG (where N is any base), and asymmetric CHH (where H = A, T, or C). The maintenance activity of MET1 can replicate CG DNA methylation even when the initial trigger for DNA methylation is genetically removed [9,10]. This may be explained in part by the fact that Dnmt1-type DNA methyltransferases have a strong preference for hemimethylated substrates, such as those left by DNA replication of a CG dinucleotide that is methylated on both strands [11]. However, non-CG DNA methylation is inherited differently and appears to require active signals to continually target regions of DNA for methylation [12]. In the case of CNG methylation, this signal seems to come from histones that are associated with the DNA. CNG methylation is mostly controlled by the methyltransferase CMT3, and also often requires histone H3 lysine 9 dimethylation (H3K9me2) by the SET domain protein SUVH4/KRYPTONITE (KYP) [13,14].

On the other hand, asymmetric methylation (which lacks an adjacent methylcytosine to provide epigenetic information after DNA replication) is mostly controlled by the DNA methyltransferase DRM2, which is targeted by 24-nucleotide short interfering RNAs (siRNAs) produced though RNA interference pathways [15–18]. *DRM2* and the closely linked gene *DRM1* encode proteins that are homologous to the mammalian de novo DNA methyltransferase Dnmt3 [19]. Notably, the *drm2* single mutant is identical to the *drm1 drm2* double mutant for all phenotypes tested [18]. siRNAs and DRM2 are also important for the initial establishment of DNA methylation in all sequence contexts, since a suite of siRNA metabolism mutants in the genes encoding nuclear RNA POLYMERASE IV *(NRPD1a)*, RNA-DEPENDENT RNA POLYMERASE2 *(RDR2)*, DICER-LIKE3 *(DCL3)*, and ARGONAUTE4 *(AGO4)* fail to establish DNA methylation at the direct repeats of the *FWA* locus when a new copy of *FWA* is transformed into plants [15].

The maintenance of non-CG DNA methylation at endogenous sequences shows very locus-specific differences in its requirement for different DNA methyltransferases [17]. For example, the direct repeat loci *FWA* and *MEA-ISR* require RNA interference (RNAi) and DRM2 for both the CNG and asymmetric DNA methylation, whereas non-CG methylation at the centromeric retrotransposon *Ta3* solely depends on CMT3. At other loci such as the small euchromatic transposon *AtSN1* and at silent alleles of the *SUPERMAN* gene, DRM2 and CMT3 act redundantly to maintain non-CG DNA methylation [17,20]. Another example of this redundancy between DRM2 and CMT3 is the fact that neither *drm1 drm2* nor *cmt3* mutants show any morphological defects, but the *drm1 drm2 cmt3* triple mutant shows a pleiotropic suite of developmental abnormalities [17]. Thus, like CG DNA methylation, non-CG DNA methylation can affect developmentally important gene expression.

We sought to understand the mechanisms that underlie propagation of non-CG DNA methylation during plant development. Here we show that non-CG methylation that controls developmental genes can be readily restored after it is lost, implying the existence of persistent targeting signals that remain in the absence of DNA methyltransferase function. We provide evidence that these signals include input from RNA silencing pathways, from the chromatin remodeling protein DRD1, and from histone methylation. We further show that DRD1 works along with the 24-nucleotide siRNA pathway in the establishment of DNA methylation, and works through both the DRM2 and CMT3 methyltransferases in the maintenance of DNA methylation. These results help to define the different mechanisms that control non-CG DNA methylation and its involvement in developmental gene regulation.

## Results/Discussion

### Inheritance of *drm1 drm2 cmt3* Developmental Phenotypes Is Strongly Correlated with the *drm2 cmt3* Genotype

**Figure 1:**
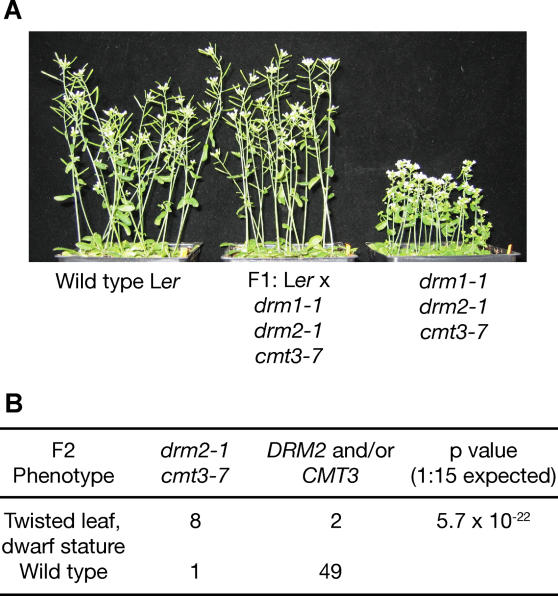
Inheritance of *drm1 drm2 cmt3* Developmental Phenotypes (A) Developmental phenotypes of *drm1–1 drm2–1 cmt3–7* are fully recessive when crossed to wild-type L*er*. (B) The developmental phenotypes of the original *drm1–1 drm2–1 cmt3–7* plants are inherited with the *drm2–1 cmt3–7* genotype in the F2 of a backcross. Phenotypes were scored blindly before the plants were genotyped. The *p-*value was calculated from a chi square test based on the null hypothesis that the developmental phenotypes were segregating independently of the *drm2–1* and *cmt3–7* genotypes, such that one would only expect 1/16th of the twisted leaf dwarf plants to be *drm2–1 cmt3–7* double mutants. The two plants scored as having a mutant phenotype that were not homozygous for *drm2–1 cmt3–7* were *drm2–1 cmt3–7/CMT3.*

It was previously shown that *drm1 drm2 cmt3* triple mutants display a pleiotropic set of developmental abnormalities [17]. Interestingly, we found that these developmental phenotypes are strongly penetrant and largely homogeneous in a population of *drm1 drm2 cmt3* plants, unlike the stochastic nature of the developmental phenotypes seen in *ddm1* and *met1* mutants. Furthermore, successive generations of inbreeding did not exacerbate the developmental phenotype of *drm1 drm2 cmt3*. *drm1 drm2 cmt3* plants in the Landsberg *erecta* (L*er*) ecotype show three major defects: a twisted leaf shape, shorter stature, and partial sterility which is evidenced by short siliques that produce fewer seeds than wild type (100% penetrance of the sterility phenotype is shown in Table S1). Flowering time in *drm1 drm2 cmt3* is similar to wild type. We found that, unlike *met1* phenotypes, all of these defects were entirely recessive when *drm1–1 drm2–1 cmt3–7* was crossed to wild type L*er* (Figure 1A). To further characterize inheritance of the *drm1 drm2 cmt3* phenotype, we selected ten plants from the F2 generation of this cross that showed a twisted leaf and short stature phenotype, and an additional 50 plants with wild-type morphology, and all were genotyped for the *drm2–1* and *cmt3–7* mutations. We did not genotype *drm1–1,* because the *drm2–1* single mutant has all of the phenotypes of *drm1–1 drm2–1,* and because the *DRM1* and *DRM2* genes are tightly linked at a distance of approximately 1 cM [18]. Twisted leaf and dwarf stature phenotypes in the F2 segregated strongly with the *drm2–1 cmt3–7* genotype (Figure 1B). Only one plant out of 50 scored with a wild-type morphology had the *drm2–1 cmt3–7* genotype, and this observation may have resulted from incomplete penetrance of the developmental phenotype. Only two plants with a twisted leaf and dwarf phenotype were heterozygous for the *cmt3–7* mutant, and thus contained a wild-type *CMT3* gene. These plants may have indeed inherited these developmental defects epigenetically, or might have been scored as dwarf due to developmental variability caused by growth conditions. Thus, in 57/60 cases tested, the predicted phenotype of the F2 plants correlated with their genotype. Overall, these F2 segregation data demonstrate a fundamental difference between developmental defects in *drm1 drm2 cmt3* and those seen in *ddm1* and *met1*—the former are generally not inherited independent of the *drm1 drm2 cmt3* genotype.

### Transforming *drm1 drm2 cmt3* with *DRM2* or *CMT3* Restores Normal Development

**Figure 2:**
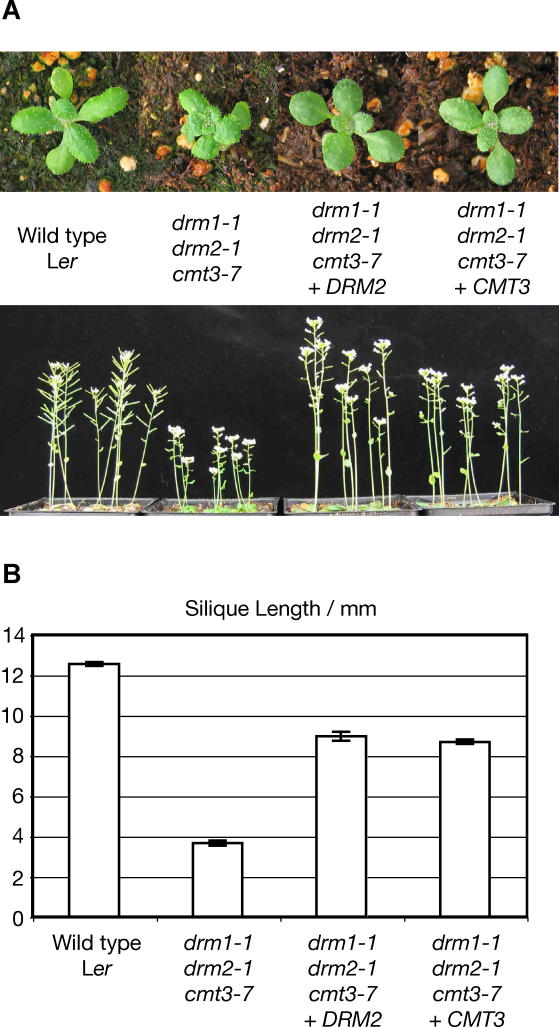
*drm1 drm2 cmt3* Phenotypes Are Efficiently Restored to Wild Type by Transformed *DRM2* or *CMT3* (A) Normal rosette leaf shape and stature are restored in *drm1–1 drm2–1 cmt3–7* transformed with either *DRM2* or *CMT3*. (B) The sterility of *drm1–1 drm2–1 cmt3–7* plants is partially restored in the T2 generation of *DRM2* or *CMT3* transformants. The length of ten mature siliques on the primary stem was measured; analysis was started at the third silique of the stem. Between eight and 36 individual plants were measured. Error bars show standard error of the mean.

In the backcross experiment shown in Figure 1A, a correctly expressed and methylated parental genome is introduced along with the wild-type *DRM2* and *CMT3* genes. This complicates interpretation of the experiment, because the genome from the wild-type parent may bring in signals that confer correct developmental regulation to chromosomes derived from the *drm1 drm2 cmt3* parent. Therefore, as a further test of whether normal development could be restored to *drm1 drm2 cmt3* mutants, we introduced either *DRM2* or *CMT3* by *Agrobacterium-*mediated plant transformation into these plants, and asked whether these transgenes could confer a wild-type morphological phenotype. Both *DRM2* and *CMT3* completely restored normal leaf shape and wild-type stature when transformed into *drm1–1 drm2–1 cmt3–7* (L*er*) (Figure 2A). Importantly, recovery of the normal phenotype in DRM2 and CMT3 transformants implies that the active signals that target non-CG DNA methylation are still present in the *drm1 drm2 cmt3* triple mutant. This restoration is consistent with a model in which *drm1 drm2 cmt3* developmental phenotypes result mostly from genes that are overexpressed when silencing-associated non-CG methylation is lost. In this scenario, these genes would be re-silenced when *DRM2* or *CMT3* are introduced by transformation. Alternatively, loss of non-CG DNA methylation might also result in inappropriately low expression of endogenous genes. For instance, the loss of DNA methylation on silencer elements could result in transcriptional suppression. The sterility defect seen in *drm1–1 drm2–1 cmt3–7* plants was greatly reduced in *drm1–1 drm2–1 cmt3–7* plants transformed with *DRM2* or *CMT3*. However, this defect was not completely reversed because the silique length in transformed plants did not reach wild-type levels in the T2 generation (Figure 2B). Failure of DRM2 or CMT3 transgenes to fully reverse the sterility defect of *drm1–1 drm2–1 cmt3–7* may reflect incomplete complementation. However, we did observe shorter siliques in multiple independent lines of both DRM2- and CMT3-transformed plants (unpublished data). These data therefore suggest that, although most of the developmental defects seen in *drm1–1 drm2–1 cmt3–7* are completely restored when wild-type DRM2 and CMT3 genes are reintroduced, the sterility defect may be to some extent inherited epigenetically.

### DRD1 Is Required for Establishment of DNA Methylation (De Novo DNA Methylation) of Transformed *FWA*

**Figure 3:**
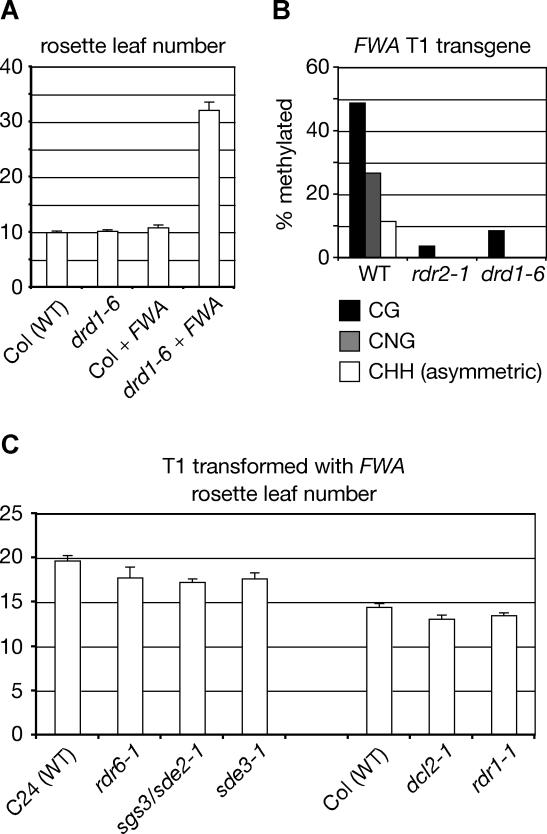
DRD1 Is Required for De Novo DNA Methylation of Tandem Repeats (A) DRD1 is required for de novo silencing of transformed *FWA*. Flowering time in untransformed and transformed T1 plants is shown—overexpression of *FWA* causes late flowering. Col, Columbia ecotype; WT, wild type. (B) DRD1 is required for de novo DNA methylation of transformed *FWA*. DNA methylation of the *FWA* transgene was measured by bisulfite genomic sequencing in T1 plants. Graph represents the percentage methylation in different sequence contexts. (C) Several RNAi mutants are competent for de novo silencing of transformed *FWA*. Flowering time for each transformed mutant is shown adjacent to its corresponding wild-type ecotype.

DRD1 is an SNF2-related ATPase and putative chromatin-remodeling protein that is required to establish and maintain RNA-directed non-CG DNA methylation triggered by a transcribed inverted repeat [21,22]. Tandem repeat sequences are probably recognized by a different mechanism than that used for inverted repeats, which have the capacity to generate double-stranded RNA by monodirectional transcription. We therefore sought to test whether DRD1 plays a role in de novo DNA methylation and silencing of transformed *FWA,* a tandem repeat–containing gene [18]. We found that, like *drm2* and RNA-silencing mutants from the 24-nucleotide siRNA pathway, including *rdr2, dcl3,* and *ago4* mutants [15], the *drd1–6* mutant flowered late after *FWA* transformation, but had no defect in silencing of the endogenous *FWA* gene, as shown by early flowering prior to transformation (Figure 3A). As predicted from their inability to silence transformed *FWA, drd1–6* plants also lacked de novo DNA methylation of the *FWA* transgene in the T1 generation (Figure 3B). These results indicate that DRD1 is essential for de novo DNA methylation of both transformed tandem repeats and targets of inverted repeat–generated siRNAs. They also suggest that DRD1 acts in concert with the RNA polymerase IV/RDR2/DCL3/AGO4 RNAi pathway to guide DRM2. We determined that de novo gene silencing was normal in the RNAi mutants *rdr6–1/sde1, sgs3/sde2–1, sde3–1, dcl2–1,* and *rdr1–1* (Figure 3C). The fact that many RNAi proteins are not required for de novo DNA methylation is further confirmation that *Arabidopsis* RNAi pathways are functionally specialized [23].

### DRD1 Acts through Both DRM2 and CMT3 to Maintain Endogenous Non-CG DNA Methylation

**Figure 4:**
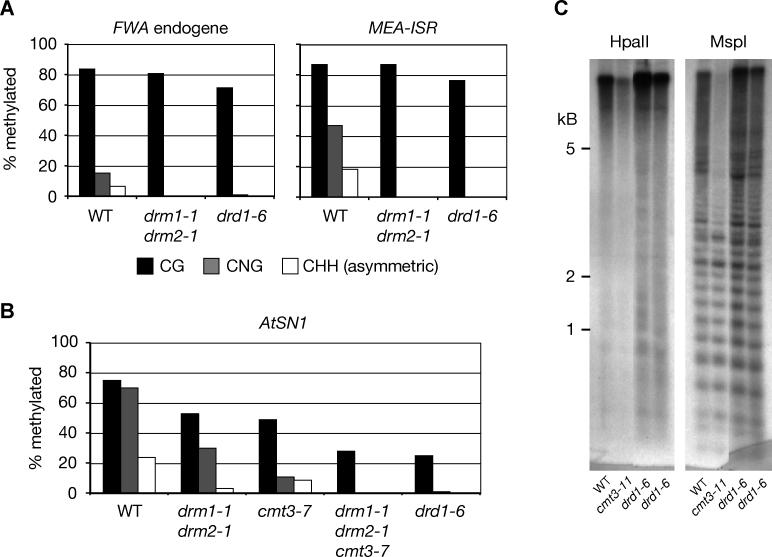
Role of DRD1 in Maintaining Non-CG DNA Methylation at Endogenous Loci (A) The *drd1–6* mutant cannot maintain non-CG DNA methylation at the endogenous direct repeats *FWA* and *MEA-ISR*. (B) *drd1–6* loses all non-CG methylation at the SINE transposon *AtSN1,* and thus phenocopies *drm1 drm2 cmt3*. DNA methylation was measured by bisulfite genomic sequencing. (C) Southern blot analysis of DNA methylation at the pericentromeric retrotransposon *Ta3*. HpaII digestion at CCGG is blocked by CG or CNG DNA methylation, whereas MspI digestion at CCGG is blocked by CNG methylation. WT, wild type.

We tested whether DRD1 acts through the DRM2 and/or CMT3 methyltransferases in its control of non-CG methylation, by testing the effect of *drd1–6* on maintenance of DNA methylation at different endogenous loci. At the endogenous direct repeats present at *FWA* and *MEA-ISR, drd1–6* lacked all non-CG methylation but did not affect CG methylation (Figure 4A). This phenotype is identical to mutants in the RNA polymerase IV/RDR2/DCL3/AGO4 RNAi pathway and *drm1 drm2* at these loci [15,17]. Importantly, at the SINE element *AtSN1,* the *drd1–6* mutant also lacked all non-CG methylation (Figure 4B). This is interesting because the *drm1 drm2* or *cmt3* mutants have only moderate effects on non-CG methylation at this locus, yet the *drm1 drm2 cmt3* triple mutant shows a loss of all non-CG *AtSN1* methylation [17]. This suggests that DRD1 can act through both DRM2 and CMT3 at endogenous genes such as *AtSN1*. Our finding is consistent with the fact that multiple mutant alleles of *drd1* were isolated from a screen for plants that could not maintain non-CG DNA methylation and transcriptional gene silencing targeted by an inverted repeat of the soybean α′ promoter [21], yet neither DRM2 nor CMT3 were identified by this screen. This suggests that like *AtSN1,* the target of α′ siRNAs has non-CG DNA methylation that is controlled by DRD1, which acts through the redundant action of the DRM2 and CMT3 methyltransferases.

The DRD1-dependent non-CG DNA methylation at *AtSN1, FWA,* and *MEA-ISR* is associated with the presence of endogenous siRNAs corresponding to these loci. In contrast, the pericentromeric retrotransposon *Ta3* lacks siRNAs, as shown by their absence from a very large small-RNA dataset compiled using the massively parallel signature sequencing technology [24]. This is also consistent with the fact that the *ago4–1* mutation had no effect on DNA methylation at *Ta3* [25]. Instead, at *Ta3,* CNG DNA methylation depends solely on the CMT3 DNA methyltransferase [17]. Importantly, *drd1–6* mutants showed no defect in CNG DNA methylation at *Ta3,* as assayed by a Southern blot with the CNG methylation–sensitive restriction enzyme MspI (Figure 4C). This can be contrasted with the *cmt3–11* mutant, in which digestion with MspI yields a far greater proportion of low-molecular weight bands consistent with restriction enzyme cleavage of DNA that lacks CNG methylation. Thus, *Ta3* is a locus where CMT3 maintains CNG DNA methylation independent of siRNAs and of DRD1.

### Developmentally Important Non-CG DNA Methylation Is Targeted by RNAi and DRD1

**Figure 5:**
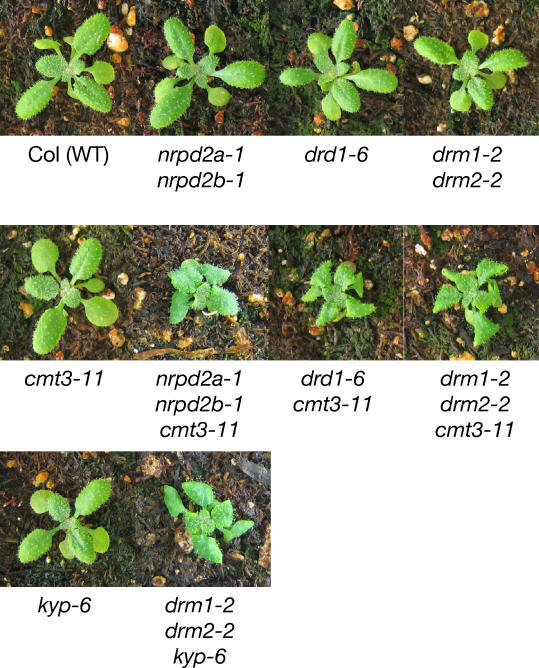
Developmentally Important Gene Regulation by DRM2 and CMT3 Requires RNAi, DRD1, and KRYPTONITE Rosette leaf shape defects in a variety of multiple mutant combinations are shown. Col (WT), wild-type Columbia ecotype.

The fact that normal development is largely restored when *DRM2* or *CMT3* are reintroduced suggests that these enzymes are actively targeted by signals that persist in the *drm1 drm2 cmt3* mutant. To test this model further, *cmt3* was combined with null mutants in the RNA polymerase IV subunit–encoding gene *NRPD2a* and in *DRD1,* both of which are required for RNAi-directed DNA methylation [21,26–28]. *NRPD2a* encodes the second largest subunit of RNA polymerase IV, which acts together with and is necessary for the activity of NRPD1a and NRPD1b [26–29]. *NPRD2b* encodes a closely related gene copy which is likely nonfunctional [27,28]. We generated a *nrpd2a-1 nrpd2b-1 cmt3–11* triple mutant utilizing T-DNA mutations all isolated in the Columbia (Col) wild-type background. We also constructed a *drd1–6 cmt3–11* double mutant in the Col ecotype. As a control, we isolated new T-DNA mutations in *DRM1, DRM2,* and *CMT3,* and constructed the *drm1–2 drm2–2 cmt3–11* triple mutant in the Col background. The Col *drm1–2 drm2–2 cmt3–11* mutant has a phenotype that is similar to that of the *drm1–1 drm2–1 cmt3–7* triple mutant in the L*er* background, with minor differences. The short stature and sterility defects were more pronounced in L*er*. However, the Col *drm1–2 drm2–2 cmt3–11* mutant has a particularly strong leaf shape phenotype, in which the apical end of the rosette leaf is folded under the blade. The Col leaf shape phenotype was 100% penetrant in a population of more than 500 homozygous *drm1–2 drm2–2 cmt3–11* plants. We found that both the *nrpd2a-1 nrpd2b-1 cmt3–11* triple mutant and the *drd1–6 cmt3–11* double mutant showed a developmental phenotype identical to that of *drm1–2 drm2–2 cmt3–11* (Figure 5). These results show that the mutations in NRPD2 and DRD1 show the same effect as mutation of DRM2 when combined with mutation of CMT3. To confirm this we also constructed both the *nrpd2a-1 nrpd2b-1 drm1–2 drm2–2* quadruple mutant and the *drd1–6 drm1–2 drm2–2* triple mutant and found that these plants had a wild-type morphological phenotype (unpublished data). These results suggest that the role of DRM2 in developmental gene regulation requires RNAi and the RNA-directed DNA methylation factor DRD1. DRD1 does not control all developmental regulation by DRM2 and CMT3, however, because the single *drd1–6* mutant has a wild-type morphological phenotype. This contrasts with *AtSN1* non-CG methylation, where *drd1–6* phenocopies *drm1 drm2 cmt3*. Since DRM2 requires DRD1 for establishment and maintenance of DNA methylation at all loci tested, we assume that CMT3 has a DRD1-independent targeting pathway, as exemplified by CNG methylation at the *Ta3* retrotransposon (Figure 4C).

### Developmentally Important Non-CG DNA Methylation Is Targeted by Histone H3K9 Methylation

The observation that the *drm1–2 drm2–2 nrpd2a-1 nrpd2b-1* plants did not show the developmental phenotypes of *drm1–2 drm2–2 cmt3–11* suggests that the control of normal gene expression by CMT3 is not solely directed by RNAi. We therefore tested whether histone H3K9 dimethylation (H3K9me2) is required to target CMT3 at developmental genes, by creating *drm1–2 drm2–2 kyp-6* plants that lack the SET domain histone methyltransferase KRYPTONITE (KYP)/SUVH4. The *kyp-6* allele is a newly isolated T-DNA allele such that the *drm1–2 drm2–2 kyp-6* plants are in a pure Col background. We found that the *drm1–2 drm2–2 kyp-6* plants displayed a very similar phenotype to *drm1–2 drm2–2 cmt3–11* (Figure 5). This suggests that the loss of KYP-mediated H3K9me2 phenocopies the loss of CMT3, when combined with mutations in *DRM* genes. Thus, both RNAi pathways and the silencing-associated histone modification H3K9me2 can target non-CG DNA methylation to developmentally important genes.

### A Model for the Targeting of Locus Specific Non-CG DNA Methylation

Our work shows that regulation of plant development by non-CG DNA methylation differs fundamentally from MET1-dependent CG DNA methylation that controls normal gene expression. Non-CG DNA methylation is directed in part by RNAi factors, in part by DRD1, and in part by histone H3 lysine 9 methylation through KYP. It is also efficiently restored after it is lost, implying that the targeting signals responsible for its propagation are persistent in plants that lack the DNA methyltransferase enzymes DRM2 and CMT3.

**Figure 6:**
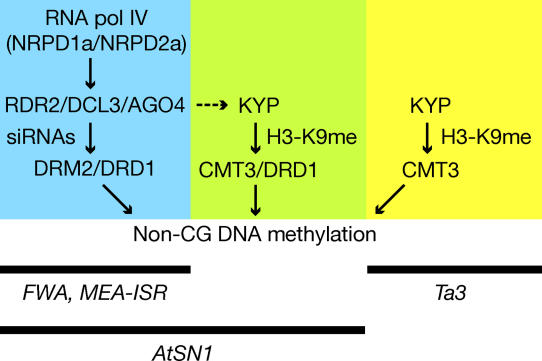
A Model for the Inheritance of Non-CG DNA Methylation in Arabidopsis thaliana Background colors represent different pathways for targeting of non-CG DNA methylation. DRM2 is guided by an RNAi pathway initiated by DNA-dependent RNA polymerase IV. CMT3 is guided by histone H3 lysine 9 dimethylation (H3K9me2) that depends on KRYPTONITE/KYP. At some loci, histone H3 methylation is targeted by RNAi (represented by dotted arrow). DRD1 controls both DRM2 and CMT3, but there is also a DRD1-independent pathway that directs CMT3.

Figure 6 presents a model for the action of several targeting pathways that control the locus-specific propagation of non-CG DNA methylation patterns. In one branch of this pathway, the 24-nucleotide siRNA pathway acts together with DRD1 to target the DRM2 DNA methyltransferase. Certain loci like *FWA* and *MEA-ISR* appear to only use this pathway, since all non-CG methylation is lost at these loci in the RNAi mutants and in the *drd1* and *drm2* mutants. Other loci, such as *AtSN1,* appear to use a combination of the RNAi/DRD1/DRM2 pathway and a second pathway, in which CMT3 is guided by histone methylation through KYP. We propose that DRD1 acts in both of these pathways, which would explain why DRD1 can facilitate non-CG DNA methylation by both DRM2 and CMT3, even though these enzymes have locus-specific effects. DRD1 is a SNF2-related ATPase (from a plant-specific subfamily), suggesting that it is a chromatin remodeling protein, and such an activity may permit DRM2 and CMT3 to methylate nucleosomal DNA in vivo. In yet a third pathway, exemplified by the *Ta3* locus, CMT3 propagates CNG DNA methylation without siRNAs or the need for DRD1.

Ultimately, we hope to understand how evolution has co-opted non-CG DNA methylation to control developmentally important endogenous genes. DNA methylation may silence developmental regulators in a tissue-specific manner, or could be a general mechanism for repressing genes that have a deleterious effect on normal development when ectopically overexpressed. In particular, it will be interesting to investigate how genes controlled by RNAi, DRD1, DRM2, KYP and CMT3 differ from those whose normal regulation requires CG DNA methylation maintained by MET1. Furthermore, as exemplified by the *FWA* gene, loss of MET1-mediated CG DNA methylation can be associated with loss of non-CG DNA methylation. This indicates that there is feedback between CG and non-CG DNA methylation, and that some genes may be regulated by both mechanisms.

During evolution, the acquisition of regulation of endogenous genes by non-CG DNA methylation may involve local sequence repeats and/or the presence of homologous small RNAs. Furthermore, proximity to transposable elements was first suggested by Barbara McClintock as a mechanism for gene regulation during maize development [30]. It is possible that non-CG DNA methylation of transposons has contributed to the evolution of development in *Arabidopsis* and in other plants.

## Materials and Methods

### Plant materials.

Plants were grown under continuous light conditions. The *drm1–1, drm2–1, cmt3–7, rdr2–1, drd1–6, rdr6–1, sgs3/sde2–1, sde3–1, dcl2–1, rdr1–1, nrpd2a-1,* and *nrpd2b-1* mutants have been previously described [18,20,23,27,31]. *drm1–1* and *drm2–1* are T-DNA alleles isolated in the Wassilewskija (WS) ecotype—both T-DNAs are predicted to disrupt essential catalytic domains. These mutations were backcrossed five times into L*er* prior to this analysis. *cmt3–7* is a point mutation isolated in the L*er* ecotype that creates a stop codon, truncating the CMT3 protein after 27 amino acids. *drm1–2, drm2–2,* and *cmt3–11* are T-DNA insertions in the predicted methyltransferase domains of *DRM1, DRM2,* and *CMT3* that would be expected to create null mutations (T-DNAs SALK_031705, SALK_150863, and SALK_148381 respectively). *kyp-6* is T-DNA SALK_041474. The phenotypes of *drm1 drm2 cmt3* in the L*er* ecotype were scored at two main stages. Twisted rosette leaf shape was scored at approximately 2 wk, prior to bolting. Short stature was scored at approximate 5–6 wk, once the plants had made the majority of their siliques.

### Transformation with *DRM2* and *CMT3*.

The *CMT3* genomic clone we used was a kind gift from Judith Bender [13]. The *CMT3*-encoding KpnI fragment was subcloned into pCAMBIA-1300 prior to transformation. The *DRM2* gene and flanking intergenic regions were PCR amplified using Pfx (Stratagene, La Jolla, California, United States) from BAC clone T15N1 with primers JP2548 5′-GTAATGGAGATAGCTTCTCAGGATTATCATTAGC-3′ and JP2549 5′-AACCAGATTGGGGCAATATACATATAGAAGAGCC-3′. The PCR product was cloned into pCR4 (Invitrogen, Carlsbad, California, United States) and sequenced. The *DRM2* gene was then cloned as an EcoRI fragment into the pCAMBIA-1300 binary vector.

### *FWA* transformation and flowering time analysis.

Transformation of *Arabidopsis* with *FWA* and flowering time analysis were performed as described [18].

### Bisulfite genomic sequencing.

Bisulfite sequencing was performed as described [15,17,25]. To create an *FWA* transgene that can be distinguished from endogenous *FWA,* we inserted an AT dinucleotide at position −780, changing a BglII site to an EcoRI site.

### Southern blotting.

Southern blotting for *Ta3* was performed as described [17].

## Supporting Information

Table S1 Silique Length/mm for the Indicated Genotypes (57 KB XLS)

### Accession Numbers

The GenBank (http://www.ncbi.nlm.nih.gov/Genbank) GeneID accession numbers for the genes and gene products discussed in the paper are *AGO4* (817246), *BAL/SNC1* (827397), *CMT3* (843313), *DCL2* (821300), *DCL3* (823508), *DDM1* (836808), *DRD1* (816136), *DRM1* (831390), *DRM2* (831315), *FWA* (828658), *MEA* (*MEA-ISR* is downstream) (839422), *MET1* (834975), *NRPD1a* (842605), *NRPD2a* (821960), *NRPD2b* (821334), *RDR1* (838044), *RDR2* (826714), *RDR6/SDE1* (824112), *SDE3* (837047), *SGS3/SDE2* (832422), *SUPERMAN* (821888), and *SUVH4/KRYPTONITE* (831244).

## Abbreviations

Col: Columbia ecotype
H3K9me2: histone H3K9 dimethylation
L*er*: Landsberg *erecta* ecotype
RNAi: RNA interference
siRNA: short interfering RNA
